# Monoarticular Hip Involvement in Pseudogout

**DOI:** 10.1155/2015/302389

**Published:** 2015-03-09

**Authors:** Figen Kocyigit, Ersin Kuyucu, Ali Kocyigit

**Affiliations:** ^1^School of Physical Therapy and Rehabilitation, Pamukkale University, Kinikli, 20070 Denizli, Turkey; ^2^Department of Orthopedics and Traumatology, Denizli State Hospital, 20010 Denizli, Turkey; ^3^Department of Radiology, Faculty of Medicine, Pamukkale University, Kinikli, 20070 Denizli, Turkey

## Abstract

Pseudogout is the acutest form of arthritis in the elderly. Although clinical manifestations vary widely, polyarticular involvement is typical mimicking osteoarthritis or rheumatoid arthritis. Monoarticular involvement is relatively rare and is generally provoked by another medical condition. There are reported cases of hip involvement by pseudogout in monoarticular form. However, all of the cases were presented as septic arthritis. In this report, we present a case of monoarticular hip involvement mimicking soft tissue abscess. We confirmed the pseudogout diagnosis after ultrasonographic evaluation of the involved hip joint and pathological and biochemical analysis of synovial fluid analysis. Diagnosis is important to avoid unnecessary medical and surgical treatment in cases of the bizarre involvement of hip in pseudogout.

## 1. Introduction

Calcium pyrophosphate dihydrate deposition (CPPD) disease is one of the most common crystal-induced arthropathies [1]. CPPD disease is the acutest form of arthritis in the elderly. The clinical manifestations of pseudogout vary widely. It can mimic osteoarthritis, gout, rheumatoid arthritis, or pseudoneuropathic arthropathy [2]. Monoarticular involvement is relatively rare. Moreover provocation by trauma, concurrent medical or surgical illness, and intra-articular hyaluronan injection are present in patients with monoarticular involvement.

Monoarticular attacks of pseudogout most often involve the knee and less often the wrist and ankle. Recently cases with isolated involvement of hip are reported [3–6]. These cases were presenting with acute hip pain [3, 4] or longstanding hip pain [6] and septic arthritis was suspected in all of the reported cases.

In this report, we present a case of monoarticular hip involvement in pseudogout presenting as soft tissue abscess on MRI. Monoarticular hip involvement in pseudogout is a rare entity and up to our knowledge this is the first case mimicking soft tissue abscess. This report also emphasizes the importance of musculoskeletal ultrasonography in the differential diagnosis.

## 2. Case Presentation

A 64-year-old male patient was admitted with right hip pain. He had pain for 2 months, but the pain aggravated in last two weeks inhibiting his night sleep. He was able to bear weight; however gait was antalgic. The patient had no previous joint involvement of this severity before. His physical examination revealed limitation of right hip movement in every direction being most prominent on internal rotation. Range of motion was painless and unrestricted on left hip. No signs of arthritis were present at other joints. There was no loss of sensation and muscle strength. Deep tendon reflexes were normative. Laboratory findings were in normal range. Leucocyte count was 7600/*μ*l according to complete blood count. Erythrocyte sedimentation rate was 2 mL/hr. Serum C-reactive protein level was 0.113 mg/dL. Total thyroidectomy was applied to the patient three months ago for thyroid nodule, and he was under thyroid hormone replacement. Parathyroid glands were preserved during surgery.

**Figure 1:**
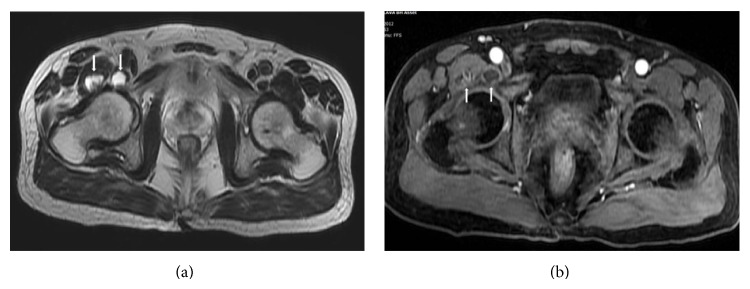
(a) Axial T2 weighted image demonstrates the periarticular collections extending into the right iliacus muscle and insertion of the psoas muscle (arrows). (b) Axial fat-saturated postcontrast T1 weighted image shows peripheral enhancement of the cystic collections mimicking abscess (arrows).

Hip MRI revealed effusion at right hip joint and cystic collections in the periarticular soft tissue were in favor of focal abscess (Figure 1). Laboratory findings were in normal range, so we decided to perform diagnostic ultrasonography. On musculoskeletal ultrasonography, there were periarticular cystic collections associated with right hip joint. We did not observe synovial hypertrophy or hyperechoic aspects in the right hip joint as defined by Filippucci et al. [7]. Ultrasonography guided cyst aspiration was performed. We aspirated three milliliters of yellow colored fluid with decreased viscosity. Cell count of the aspirated fluid documented 0.03 K/*μ*l white blood cells, 0.01 M/*μ*l red blood cells, and 9 K/*μ*l thrombocytes. Differentiation of white blood cells was as follows: lymphocytes 31.9%, neutrophils 19.8%, monocytes 18.7%, eosinophils 4.7%, and basophils 4.7%. We injected triamcinolone acetonate (40 mg) into the lesion after aspiration. Direct examination of the fluid revealed birefringent crystals under polarized light microscopy. We diagnosed the patient as pseudogout according to pathological examination of the synovial fluid. The pain of the patient dramatically resolved after aspiration and injection. Etodolac 200 mg/day was prescribed. Patient is under regular follow-up for one year and did not experience similar attack at any joints.

## 3. Discussion

Pseudogout may present with many complex clinical phenotypes. Estimates from prevalence studies indicate that it is less than 10% [5]. According to the proposed diagnostic criteria, for pseudogout, demonstration of CPPD crystals obtained by biopsy or aspirated synovial fluid by definitive means is needed for definite diagnosis [2]. The case we presented fulfills the definite diagnosis criteria.

The pathogenesis of pseudogout remains unclear. Inorganic pyrophosphate (PPi) is a potent inhibitor of mineralization where inorganic phosphate (Pi) promotes mineralization. CPPD crystals are formed when the ratio is less than 3 [8, 9]. Despite the progress in understanding of molecular mechanisms much remains to be investigated [10].

Pseudogout can be idiopathic, familial, or associated with systemic metabolic disease (hyperparathyroidism, dialysis-dependent renal failure, hypomagnesemia, and hemochromatosis) [2]. Our patient had a history of partial thyroidectomy for thyroid nodules. However, evidence from controlled studies suggests that thyroid status is not associated with increased prevalence of pseudogout [11, 12].

Monoarticular involvement of the hip joint is rare. Minor trauma, current medical or surgical conditions (pneumonia, myocardial infarction, and pregnancy), parathyroidectomy, and parenteral bisphosphonate use may trigger monoarticular involvement [2]. None of these provocative situations were present in our case.

Acute monoarticular involvement can be associated with chills, fever, systemic leukocytosis, and elevated erythrocyte sedimentation rate [2]. Despite MRI scans in favor of soft tissue abscess, the acute phase reactants and leucocyte count were in normal range.

There are reported cases of monoarticular hip involvement in pseudogout. Hamilton presented a case with longstanding hip pain that was diagnosed as pseudogout after arthroscopy [5]. Dala-Ali presented an HIV-infected case with hip pain [3]. Presenting symptoms of this patient were matching with septic arthritis. However, CPPD crystals were documented with synovial fluid analysis. The current treatment for HIV infection or the HIV infection itself may be provocative for acute pseudogout attack in the aforementioned case. Mukhopadhyay reported another case with hip involvement presenting as septic arthritis [4]. The patient was diagnosed as pseudogout after synovial fluid analysis. The authors pointed out that septic arthritis should be excluded first in acute-onset hip pain in elderly patients. They recommended clinicians performing minimal invasive diagnostic procedures instead of rushing patient into the theater. We agree with their recommendations. In our case, we diagnosed and treated the patient conservatively and successfully after aspiration of the periarticular cyst under ultrasonography guidance. If the magnetic resonance imaging findings matching with local abscess were taken into account unnecessary antibiotic use and/or surgical abscess drainage could have been applied.

Treatment options for pseudogout are nonsteroidal anti-inflammatory agents, systemic or intra-articular corticosteroids, adrenocorticotropic hormone, and prophylactic low-dose colchicine [2]. We used intra-articular corticosteroid and nonsteroidal anti-inflammatory agents for the treatment of the presented case. The symptoms resolved dramatically. Patient is under follow-up without any attacks or other joint involvement of pseudogout.

Pseudogout is one of the common entities causing arthralgia and arthritis in the elderly. Ramonda et al. reported the prevalence of chondrocalcinosis to be 10.4% in older Italians [13]. However, monoarticular involvement is rare and may present with septic arthritis symptoms [3, 4]. Up to our knowledge, this is the first case of pseudogout presenting as soft tissue abscess on MRI. We used diagnostic ultrasonography for differential diagnosis and documented rare monoarticular involvement of pseudogout after synovial fluid analysis.

This case also documents the efficacy of musculoskeletal ultrasonography not only in the differential diagnosis but also in microinvasive treatment. Despite being highly sensitive in soft tissue disorders, MRI would otherwise lead to unnecessary use of antibiotics and surgical interventions. Pseudogout should always be kept in mind in elderly patients presenting with monoarticular symptoms.
